# Using pseudo-labeling to improve performance of deep neural networks for animal identification

**DOI:** 10.1038/s41598-023-40977-x

**Published:** 2023-08-24

**Authors:** Rafael E. P. Ferreira, Yong Jae Lee, João R. R. Dórea

**Affiliations:** 1 Department of Animal and Dairy Sciences, University of Wisconsin-Madison, Madison, WI 53706 USA; 2 Department of Computer Sciences, University of Wisconsin-Madison, Madison, WI 53706 USA; 3 Department of Biological Systems Engineering, University of Wisconsin-Madison, Madison, WI 53706 USA

**Keywords:** Image processing, Machine learning

## Abstract

Contemporary approaches for animal identification use deep learning techniques to recognize coat color patterns and identify individual animals in a herd. However, deep learning algorithms usually require a large number of labeled images to achieve satisfactory performance, which creates the need to manually label all images when automated methods are not available. In this study, we evaluated the potential of a semi-supervised learning technique called pseudo-labeling to improve the predictive performance of deep neural networks trained to identify Holstein cows using labeled training sets of varied sizes and a larger unlabeled dataset. By using such technique to automatically label previously unlabeled images, we observed an increase in accuracy of up to 20.4 percentage points compared to using only manually labeled images for training. Our final best model achieved an accuracy of 92.7% on an independent testing set to correctly identify individuals in a herd of 59 cows. These results indicate that it is possible to achieve better performing deep neural networks by using images that are automatically labeled based on a small dataset of manually labeled images using a relatively simple technique. Such strategy can save time and resources that would otherwise be used for labeling, and leverage well annotated small datasets.

## Introduction

Computer vision (CV) systems have great potential to generate precise high-throughput phenotyping in several domains, such as precision medicine, crop and animal breeding, and farm management. Deep neural network algorithms are the state-of-the-art in such computer vision tasks and they often require large amounts of data to achieve satisfactory performance^1^. Supervised learning tasks require the training data to be annotated and most image data generated by CV systems in agriculture are not automatically annotated or easy to annotate. Additionally, such CV systems have the potential to generate large amounts of data that are labor- and resource-intensive to organize, annotate, and analyze. Objects of interest in agriculture setups are usually challenging to be manually annotated by humans, such as individual crops in a farm plot or individual animals in a herd, resulting in an even more laborious and time-consuming annotation process which is often prone to human error.

Several techniques have been proposed in the past decades attempting to enable deep neural networks to learn from small datasets, reducing costs related to data collection, annotation, and preprocessing while maintaining good predictive performance. Such trend can be noticed through the transition from strictly supervised approaches with large, annotated datasets to approaches that use partially annotated or unlabeled data that require less or no annotation. Among those techniques, there have been great advances in the field of few-shot learning^2^, and more notably semi-supervised learning (SSL)^3^.

In livestock systems, animal identification is the first step for individual animal phenotyping. Current state-of-the-art computer vision methods for animal identification usually require large labeled datasets that can be labor-intensive to annotate. ^4^And^5^ trained convolutional neural networks (CNNs) to identify Holstein cows using top-down view images of their back^6^, used detection and classification CNNs for face detection and recognition of individual Holstein cows,^7^ used RGB-D (Red, Green, Blue, Depth) images to identify Holstein cows and estimate their body condition score, and^8^ proposed their own 2D CNN to individually identify pigs using images of their faces. Although such studies focused on a closed-set problem, which involves identification within a fixed group of animals, this scenario is not commonly encountered in commercial farms where the animal movement in and out of the herd is highly dynamic. Consequently,^9^ introduced a novel approach to address the challenge of identifying individuals in an open-set scenario. Their method utilizes deep metric learning to generate image representations that exist within a latent space that facilitates the clustering of images belonging to the same animal, allowing for individual identification even in dynamic environments. Nevertheless, it remains unclear how these proposed methods would effectively scale for application in large commercial herds. This includes not only evaluating their predictive performance but also addressing the operational challenges associated with data collection, processing, and annotation.

As previously mentioned, existing studies on animal identification have predominantly focused on fully supervised approaches that require extensive image annotation for both closed-set and open-set scenarios, which can be labor-intensive and time-consuming. In this context, semi-supervised learning can be an effective tool for leveraging unlabeled data collected from camera systems installed at farms that would otherwise require significant human effort to annotate. In SSL, the machine learning algorithm learns structured information from the labeled portion of the dataset and uses the patterns captured from the unlabeled data to improve its predictive performance and generalization power. Thus, scenarios where labeling all the data available is too expensive or even unfeasible, but it is still possible to label part of the dataset, are the most adequate for SSL. In the context of livestock systems,^10^ introduced an SSL method for teat-end condition classification on dairy cows and found a significant improvement in performance by taking advantage of unlabeled data through their proposed algorithm. However, applications of SSL for individual animal identification using computer vision are yet to be explored.

Within SSL, a popular technique is pseudo-labeling, which consists of iteratively including confident predictions of unlabeled data into the training dataset^11^. Pseudo-labeling allows for a simple and effective way to improve the predictive performance of trained machine learning models when labeling more data is costly and large amounts of unlabeled data are available. Pseudo-labeling can be easily implemented with various machine learning algorithms applied to different datasets (if unlabeled data is available) and domains, including applications in agriculture^12,13^, medicine^14^, person re-identification^15^, and remote sensing^16^ for example. The simplicity and versatility of pseudo-labeling were our main motivations for evaluating the application of this technique for training deep neural networks for animal identification.

The objective of this study was to evaluate the potential of a semi-supervised learning technique called pseudo-labeling to improve the predictive performance of deep convolutional neural networks trained to identify individual Holstein cows using labeled training sets of varied sizes and a larger unlabeled dataset. The core emphasis of this work was not on introducing a novel SSL method, but rather to address a biological problem—the identification of individual animals, and to present a fresh perspective to approach this issue—that of semi-supervised learning. Thus, we focused on studying pseudo-labeling in the novel setting of animal identification, rather than proposing extensive modifications to current semi-supervised methods. The method evaluated in this study is complementary to current animal identification research, as it can be seamlessly applied to previously trained models without requiring any modifications in the model architecture or optimization procedure.

## Material and methods

All the animal procedures were approved by the University of Wisconsin-Madison, College of Agricultural and Life Sciences, Animal Care and Use Committee (IACUC #A006270-R01). The experiment was conducted in accordance with relevant guidelines and regulations. The authors complied with the ARRIVE guidelines (https://arriveguidelines.org/).

### Data collection

Images from 59 lactating cows were taken using four Intel RealSense D435 depth cameras^17^ installed at the milking parlor exit lanes of the Emmons Blaine Dairy Cattle Research Center (Arlington, WI) between August 8th and October 7th, 2020. Top-down view images were captured twice a day following each milking sessions, triggered by cow presence detection within camera range. Because the cameras contained a depth sensor, the method for detecting a cow under the camera consisted of checking if a region inside the lane had an average distance from the camera below a certain threshold. For this study, we used a threshold value of 3 m, meaning that a snapshot would be taken only if there was an object less than 3 m away from the camera. Given that the cameras were installed at 3.5 m high, snapshots were taken if and only if there was a cow under the camera. In total, 23,709 snapshots were used in this study, of which 4695 were labeled with the corresponding cow identification code, and 20,194 were kept unlabeled. The labeled snapshots were split into training, validation, and test sets according to the capture date, as shown in Table 1. The validation set was used to define the best threshold values for each round of the pseudo-labeling algorithm (see details in “Pseudo-labeling”), and the test set worked as a final independent performance assessment.

**Table 1:** Capture dates and total number of images contained in each dataset split.

Dataset split	Initial date	Final date	Number of images
Training	August 8th	August 9th	2354
Validation	August 10th	August 20th	1161
Test	September 2nd	October 7th	1180
Unlabeled	August 21st	September 1st	20,194

Care was taken to ensure that images contained in the training, validation, and test sets were collected in different days, simulating a realistic scenario where a model is trained on certain dates and its accuracy is tested on future dates.

### Data preprocessing

**Figure 1:**
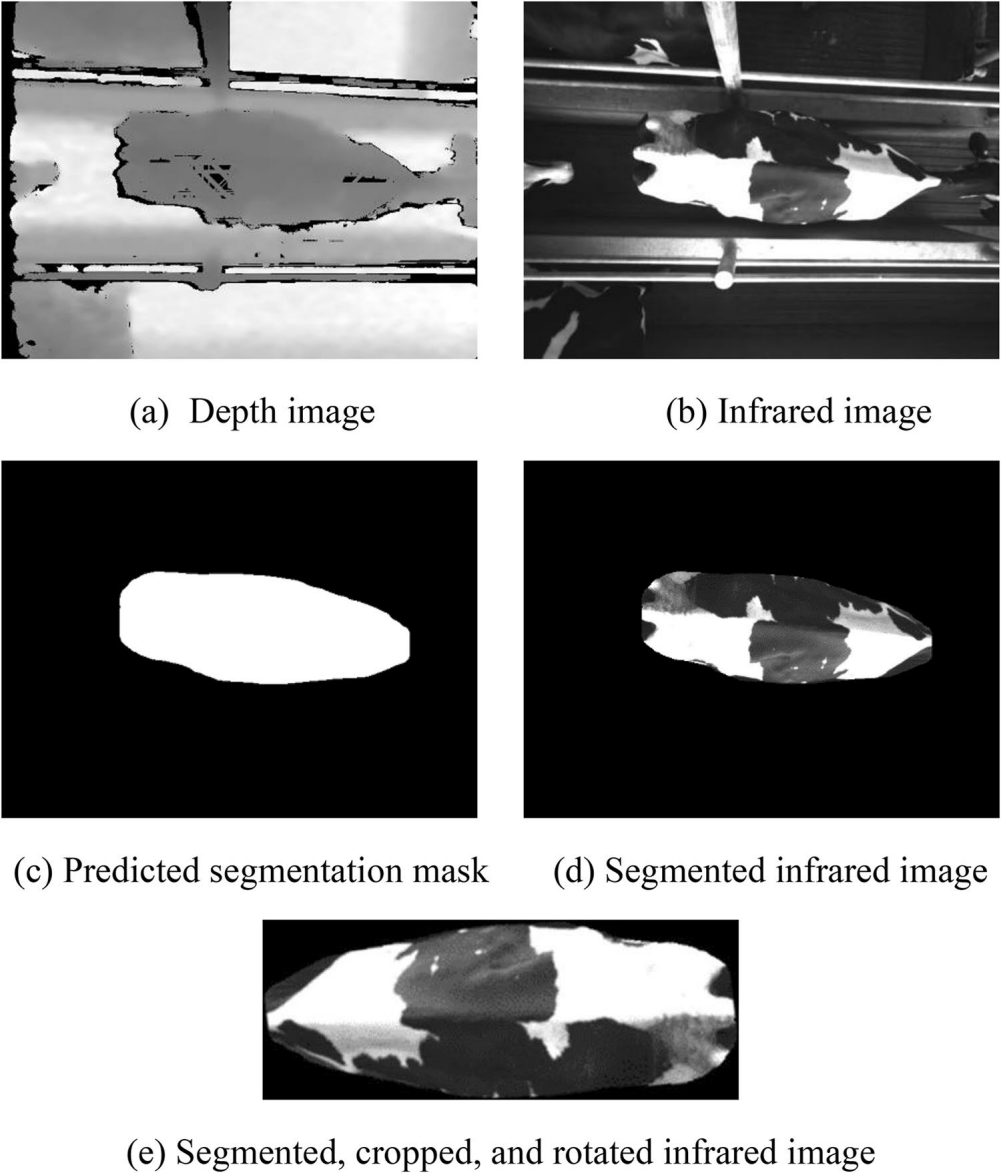
Example of a snapshot after each preprocessing stage. (**a**) Shows the original captured depth image; (**b**) shows the original captured infrared image; (**c**) shows the predicted segmentation mask generated from the trained Mask R-CNN algorithm; (**d**) shows the segmented infrared image, after applying the predicted segmentation mask to the original infrared image; and (**e**) shows the resulting image from cropping and rotating (**d**) to only contain the area around the cow.

Each snapshot consisted of a depth and an infrared image, both with a resolution of 640 × 480 pixels. The depth image contained, for each pixel, the distance in millimeters from the object in that pixel to the camera sensor, and the infrared image contained a value between 0 and 255 for each pixel, ranging from black to white, respectively. An image segmentation algorithm based on Mask R-CNN^18^ with ResNet-50^19^ as the backbone architecture was trained using a dataset of 843 depth images and the corresponding manually defined segmentation masks of cows and calves in dairy farms. This trained cow segmentation algorithm was applied to the depth images to generate segmentation masks for each snapshot, which were then used to remove all background pixels from the infrared images (i.e., pixels that did not contain the cow’s body, from tail to neck). Finally, the segmented images were cropped to include only the area containing the cow body and rotated to adjust the cow to a horizontal position. All further experiments used segmented infrared images. See Fig. 1 for an example of the preprocessing step applied to each snapshot.

### Neural network training

All neural networks in this study were trained using the Keras^20^ library available in Python, with TensorFlow^21^ as the backend. For the full iteration of pseudo-labeling trained in four rounds (see details in Pseudo-labeling), the neural networks followed the Xception architecture^22^. We have also evaluated two additional architectures for one round of pseudo-labeling—MobileNetV2^23^, and NASNet Large^24^. We selected these architectures purposefully to represent a broader spectrum of the design philosophies in deep learning—with MobileNetV2 being a lightweight and efficient design suitable for mobile and embedded vision applications; NASNet Large, a modern and high-performing architecture developed through neural architecture search; and Xception, an architecture that uses depthwise separable convolutions to enhance model efficiency, and it represents a good trade-off between model complexity and predictive performance. We compared the performance of each neural network architecture after one round of pseudo-labeling to study how such design decisions interact with the SSL approach. However, it is important to underline that our main objective was not to comprehensively compare deep learning architectures per se, but to better understand the impact of using pseudo-labeling for enhancing existing animal identification models.

All networks were trained with Transfer Learning using the ImageNet dataset^25^, meaning that the weights from all the layers except for the last two Fully-Connected (FC) layers were initialized with the values from the original corresponding networks trained using ImageNet. This technique accelerates the training process as it allows the neural network to retain the knowledge previously learned from training using a large generic image dataset.

During training, image augmentation was performed using the built-in image augmentation functionality from Keras, with the following parameters: *zoom_range* = *0.1, brightness_range* = *(0.2, 1.5), horizontal_flip* = *True, vertical_flip* = *True, fill_mode* = *‘nearest’*. This means that, during the training procedure, all training images were randomly zoomed in or out by up to 10%, had the pixel brightness adjusted to a random value between 20 and 150% of the original, and had a 50% chance of being flipped horizontally or vertically.

For each neural network, the training process was performed in two stages: feature extraction and fine-tuning. In the feature extraction stage, the neural network was trained for 30 epochs with only the weights from the last two FC layers unfrozen, keeping all other weights (i.e., the ones learned from ImageNet) unchanged. Then, in the fine-tuning stage, weights from earlier layers were unfrozen and the network was trained for 60 epochs with a smaller learning rate, allowing the network to adjust its weights to our more specific datasets and tasks. The weights were optimized using the Adam algorithm^26^ with a learning rate of 1 × 10^–3^ in the feature extraction stage and 1 × 10^–5^ in the fine-tuning stage.

### Pseudo-labeling

**Figure 2:**
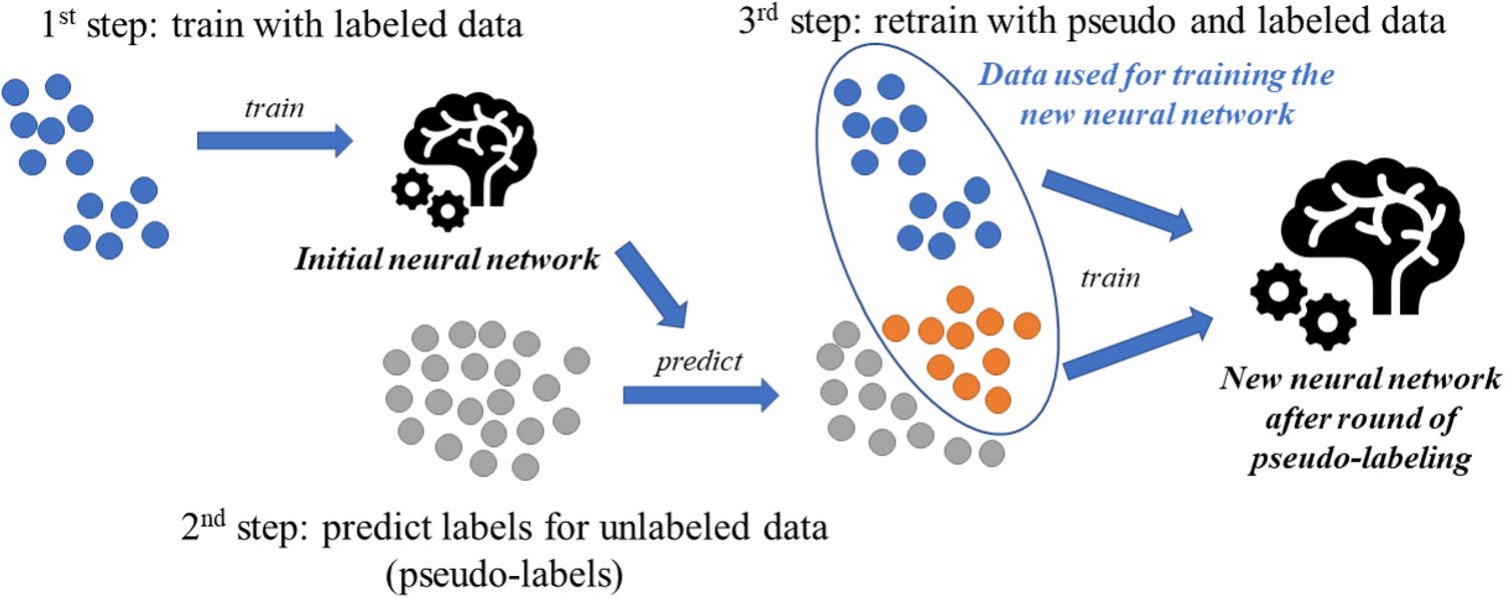
One round of pseudo-labeling, comprising of: training an initial neural network; running predictions on unlabeled data; and training a new neural network using both initial labeled data and unlabeled data with confident predictions, using the corresponding predicted classes as labels (pseudo-labels). Blue points correspond to labeled data, gray points correspond to unlabeled data, and orange points correspond to originally unlabeled data whose prediction confidence is greater than a given threshold. In the third step, such unlabeled images (orange points) are assigned their predicted classes as labels, and are added to the training set for training a new neural network.

The technique explored in this study consists of training a convolutional neural network in multiple “rounds”, with each round consisting of the following steps: first, an initial training set labeled by humans is used to train a neural network for cow identification; then this trained network performs predictions on a larger unlabeled dataset; and finally, the unlabeled images with confident predictions are added to the training set containing previously labeled images for training a new neural network. A confidence threshold value controls which unlabeled images are included in the training set for the next round, such that only images with a prediction confidence above that threshold are included. Thus, the threshold value works as a trade-off between training set size and pseudo-label quality, dictating whether the next round will contain more images with uncertain predicted labels, or fewer but more certain image labels. The new neural network is trained using both the original manually labeled dataset and the portion of the unlabeled dataset for which the prediction probabilities were above the defined threshold. After that, another round of predictions is performed on the remaining unlabeled data, and the new images and corresponding predictions are included in the next pseudo-labeling round. This process is repeated until a given stopping condition is achieved. For this study we performed up to four rounds of pseudo-labeling for each experiment. Figure 2 illustrates the steps that compose one round of pseudo-labeling.

It is important to note the difference between the technique explored in this study and the one proposed by^11^ proposes that labeled and unlabeled data are used simultaneously during the training schedule and that the pseudo-labels are recalculated after every weight update. Alternatively, in this study we perform multiple rounds of training, including new unlabeled data with the corresponding predicted labels only after full training schedules. We chose this method so that we could define a threshold value after each training round to only include unlabeled images with higher probabilities, as opposed to including every unlabeled image in the entire training procedure. Additionally, the method used in this study can be seamlessly applied to previously trained networks without any modifications in the original network architecture or optimization procedure, which might prove useful as a complementary, additional step for enhancing current animal identification networks. Part of our experiments consisted of finding the best threshold values based on the initial labeled training set.

### Experiments

We performed four types of experiments to evaluate the best scenarios for applying pseudo-labeling for animal identification using deep neural networks. Such experiments consisted of (1) varying the confidence threshold for a prediction to be included in the next training step, (2) evaluating different neural network architectures for one round of pseudo-labeling, (3) performing multiple rounds of pseudo-labeling for one of the architectures, and (4) evaluating the effectiveness of this technique with varying manually labeled initial training set sizes.

#### Threshold values

When performing pseudo-labeling, a confidence threshold value must be defined to dictate which unlabeled images and their corresponding predictions are included in the training set for the next training round. This confidence threshold is applied over the predicted confidence values generated by the trained neural network for each new image. The last layers of the deep neural networks utilized in this study contained a softmax activation function with the number of output units corresponding to the number of classes (in this case, one class for each animal, resulting in 59 units). This means that the softmax function, with the formula described in Eq. (1), was applied to the output of such networks, resulting in output values between 0 and 1 for each class, and the total sum of all output values equaling exactly 1. For that reason, the output of a neural network that contains a softmax activation function in its last layer can be interpreted as the confidence value that the network believes a given data point belongs to each class.

1 $$\sigma \left( {\varvec{z}} \right)_{i} = \frac{{e^{{z_{i} }} }}{{\mathop \sum \nolimits_{j = 1}^{K} e^{{z_{j} }} }}$$

***z*** is the *K*-dimensional vector of the network before applying softmax, *i* and *j* are class indices, *e* is Euler’s number, and *K* is the total number of classes.

The threshold values evaluated in this study were 0 (meaning that all unlabeled data would be included in the next training set), 0.5, 0.75, 0.90, 0.95, 0.98, 0.99, 0.995, 0.999, 0.9999, 0.99999, 0.999999, and 0.9999999. These values were chosen based on an analysis of the output confidence values generated by the initial trained network on unlabeled images. The implementation of the neural networks in this study allowed for output values represented by a 64-bit floating-point variable in Python ranging from 0 to 1, which can accommodate real numbers with 7 decimal digits or more, thus allowing for all threshold values chosen to be relevant. It is important to note that optimal threshold values for pseudo-labeling are highly dependent on the machine learning algorithm used and its possible output values.

#### Different neural network architectures

Aiming to study the impact of different architectural design philosophies on the use of pseudo-labeling for this particular problem, we evaluated three neural network architectures for one round of pseudo-labeling—Xception, MobileNetV2, and NASNet Large. We chose a variety of architectures that have varying number of parameters and design paradigms; however, it was not within the scope of this work to perform a comprehensive comparison of deep neural network architectures.

#### Multiple rounds

For the Xception architecture, we evaluated the impact of performing multiple rounds of pseudo-labeling. We chose to further explore the technique on such architecture because it provided a good trade-off between predictive performance, number of parameters, and training time. For each pseudo-labeling round, the best threshold value was chosen based on the accuracy on the validation set, with potentially different optimal threshold values being selected on each round. After four rounds of pseudo-labeling, we evaluated the predictive performance of the final resulting Xception model on the test set, and compared it with that achieved by the original model (trained using only manually labeled images) on the same test set.

#### Initial labeled training set size

In order to evaluate how the initial proportion of labeled and unlabeled images affects the final achieved accuracy after performing multiple rounds of pseudo-labeling, we generated random reduced versions of the initial manually labeled training set. Datasets containing 10%, 25%, 50%, 75%, and 90% of the manually labeled training set were generated using random sampling and used as initial training sets for four rounds of pseudo-labeling each. The generated datasets contained 235, 588, 1177, 1765, and 2118 manually labeled images, respectively, which resulted in labeled proportions of 1%, 3%, 5%, 8%, and 9%. For this experiment, the Xception architecture was evaluated for four pseudo-labeling rounds.

### Evaluation metrics

Each threshold value generated a different neural network, trained using both the labeled images and the unlabeled images whose prediction confidences surpassed the confidence threshold. For each pseudo-labeling round, the best threshold value was the one that generated the neural network that achieved the highest accuracy on the validation set (consisting of 1161 images). Both the baseline neural network (trained using just manually labeled images) and the models generated after each pseudo-labeling round were evaluated on an independent test set containing 1180 images that were not included in the training or the validation sets, in order to assess the performance improvements achieved from performing pseudo-labeling. For each model, both the accuracy and the Mean Average Precision (mAP) on the test set were calculated. Accuracy corresponds to the proportion of correctly classified images over the total number of images in the test set, and mAP corresponds to the micro-average of the area under the precision-recall curve for each class, averaged over all classes. Accuracy was used as the main performance metric because both validation and test sets were balanced, meaning that the number of images per class was the same for all classes, and mAP was calculated to allow for comparison with previous related work in dairy cattle identification.

## Results and discussion

The baseline Xception model, trained using the initial labeled training set containing all 2354 images, achieved an accuracy of 83.45% on the validation set and 77.54% on the test set. As described in *Evaluation metrics*, the calculated mAP was 90.48% on the validation and 85.23% on the testing set. Although the performance on the validation set was slightly higher than on the testing set, there is no direct explanation for that difference as the images from both validation and testing sets were taken on different days from those in the training set, as previously explained in *Data collection*. The baseline MobileNetV2 and NASNet Large models achieved accuracies of 81.55% and 85.00% on the validation set, and 75.00% and 77.03% on the test set, respectively. These findings were consistent with our expectations, given the differences in model architecture and parameter count. MobileNetV2, being a lightweight model with 3.5 million parameters, NASNet Large, a larger model with 88.9 million parameters, and Xception, which falls in between with 22.9 million parameters, demonstrated the well-known trend that models with higher parameter counts tend to exhibit greater predictive performance.

In our study, hyperparameter tuning was not performed for training the neural networks, since the network architectures were the same as in the corresponding papers, and the weights were initialized using Transfer Learning. Still, we generated a validation set to choose the best threshold values for each round of the pseudo-labeling algorithm. Because of that, we compared the predictive performance of the baseline models (trained using the 2354 labeled training set images, before any pseudo-labeling was performed) on the validation set with other previously published studies using computer vision to identify Holstein cows. ^27^Found an accuracy of 90.55% using side-view images of 30 cows;^4^ found a Mean Average Precision of 86.07% for individual identification among 89 cows;^28^ found an accuracy of 97.01% among 45 cows; and most recently,^5^ achieved an accuracy of 98.67% using top-view images of 48 cows. These results indicate that the performance of our baseline models agreed with other similar studies that used computer vision to identify Holstein cows based on their coat color patterns.

**Figure 3:**
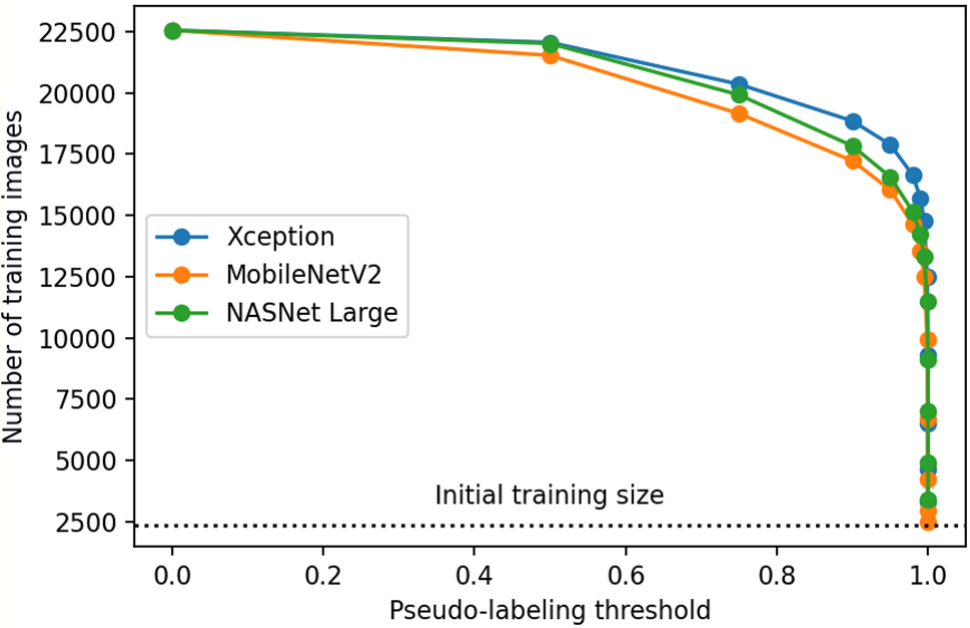
Number of resulting training images after filtering unlabeled data predictions using different threshold values. Threshold values were set to 0, 0.5, 0.75, 0.90, 0.95, 0.98, 0.99, 0.999, 0.9999, 0.99999, 0.999999, 0.9999999. Unlabeled images are filtered based on the prediction confidence resulting from the trained baseline model, which corresponds to the highest value in a neuron from the output layer after applying the softmax function. Higher threshold values restrict the images used in the next training round to only those that contain pseudo-labels with higher confidence, decreasing the training set size but potentially increasing the quality of the pseudo-labels.

**Figure 4:**
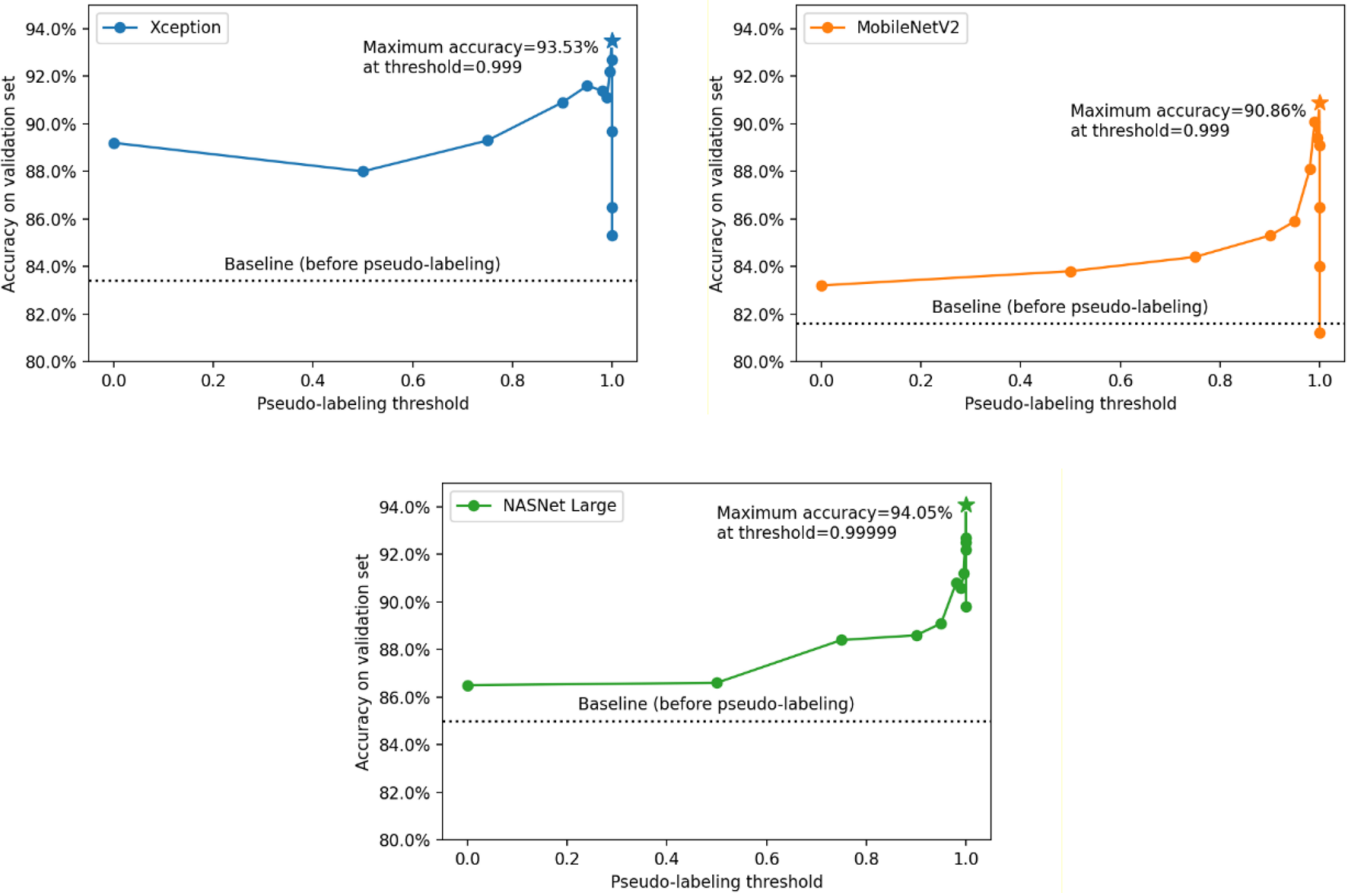
Validation set accuracy of the neural networks following each evaluated architecture, trained using images filtered based on different confidence threshold values. Threshold values were set to 0, 0.5, 0.75, 0.90, 0.95, 0.98, 0.99, 0.999, 0.9999, 0.99999, 0.999999, 0.9999999. The best models for each architecture, represented with stars, were trained using both manually labeled images and unlabeled images (and their corresponding predicted labels) with confidence predictions above the optimal thresholds using the corresponding baseline model. Finding the best threshold value is key to the success of applying pseudo-labeling, as lower threshold values tend to add too many noisy (and possibly wrong) pseudo-labels, and higher threshold values tend to excessively restrict the addition of unlabeled data, approaching the results achieved with the baseline model.

On the first round of pseudo-labeling as described in *Pseudo-labeling*, the total number of images used for training decreased almost exponentially as the threshold value approached 1. This phenomenon persisted across all three evaluated architectures, as shown in Fig. 3. Lower threshold values add more images to the next training round, however, with more uncertainty on the pseudo-labels. Conversely, higher threshold values restrict the images used in the next training round to only those that contain pseudo-labels with higher confidence, decreasing the training set size but potentially increasing the quality of the pseudo-labels. As shown in Fig. 4, the accuracy on the validation set starts increasing as the threshold increases, until it reaches a maximum value at 0.999 (for Xception and MobileNetV2) or 0.99999 (for NASNet Large), and then starts decreasing as the threshold value increases further. These results reveal how one can adjust the threshold value to control the trade-off between the number of images used for training and the pseudo-label quality of the previously unlabeled images added after pseudo-labeling. Finding the threshold value that optimizes this trade-off is key for achieving the best results when using this pseudo-labeling technique.

For each architecture, we evaluated the best achieved test accuracy after one round of pseudo-labeling and the average training times on the same NVIDIA GeForce RTX 2080 GPU. Results are shown in Table 2.

**Table 2:** Best predictive accuracy, times to train the baseline model, and the minimum and maximum training times for the first round of pseudo-labeling for each architecture.

Architecture	Baseline training time (minutes)	Minimum training time (minutes)	Maximum training time (minutes)	Baseline test accuracy (%)	Best test accuracy (%)
Xception	170	224	1350	77.5	90.2
MobileNetV2	146	132	953	75.0	85.7
NASNet Large	200	331	2341	77.0	91.3

The Xception architecture provided a good trade-off between predictive and computational performance, so we decided to further investigate only this architecture in the subsequent experiments.

**Figure 5:**
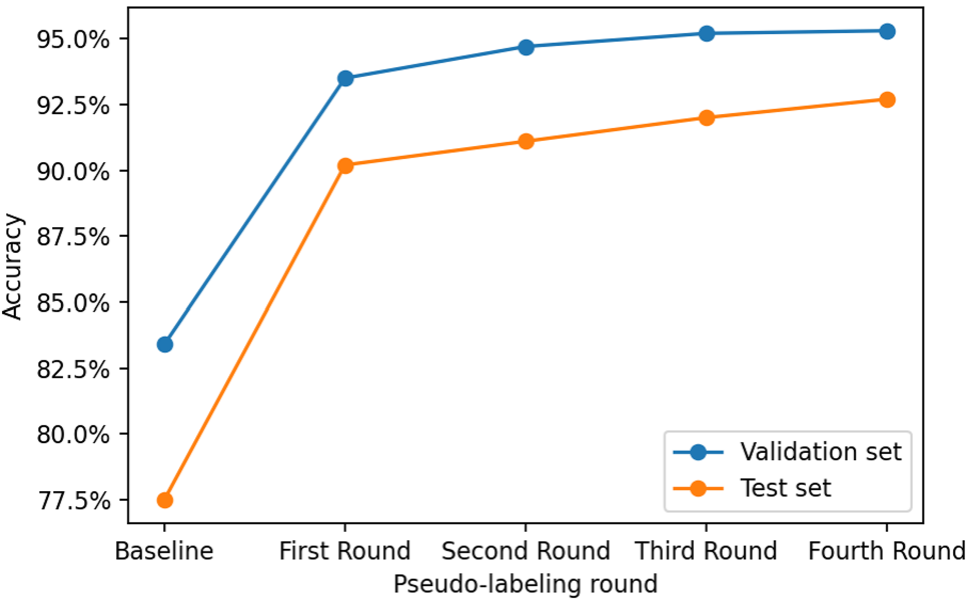
Accuracy on the validation and test sets of the trained networks after one, two, three, and four rounds of pseudo-labeling using the best threshold values in each round. The performance increases considerably after a single round of pseudo-labeling and remains roughly steady after the subsequent rounds.

^11^proposed the inclusion of an alpha hyperparameter which dictates the relative weight of the unlabeled portion of the data on the loss function value calculated at each training iteration. This process is equivalent to Entropy Regularization, and the choice of alpha controls the trade-off between giving more importance to unlabeled or labeled data during each stage of training. The rationale for defining a schedule for alpha is that in earlier epochs its value should be low, allowing the network to learn mostly from labeled data, and as the network becomes more proficient throughout training, the alpha value can be adjusted to higher values to allow for unlabeled data to be included in training with potentially more accurate predictions. This procedure uses every image from the unlabeled dataset, attributing a weight to the importance of the entire unlabeled dataset during training. Conversely, the confidence thresholding technique utilized in this study allows for a more discriminating choice of unlabeled images to be used in subsequent training rounds, completely excluding part of the unlabeled dataset, but simultaneously weighting labeled and unlabeled images the same during training.

^29^evaluated multiple SSL methods, focusing exclusively on those which consist of adding an additional loss term during training. The methods assessed in their study were either based on Consistency Regularization (Π-Model, Mean Teacher, and Virtual Adversarial Training), or Pseudo-labeling. Their implementation of Pseudo-labeling was like that proposed by Lee^11^ in the sense that unlabeled and labeled data were used in training simultaneously. However, ^29^did not discuss the use of an alpha hyperparameter, and instead incorporated thresholding, with a fixed value of 0.95 as found during their hyperparameter tuning procedure.

Since the Xception architecture provided the best trade-off between predictive and computational performance, we decided to only use this architecture for further experiments. For the Xception architecture, the best threshold value (i.e., the one that maximizes validation accuracy) in the first round of pseudo-labeling was found to be 0.999. The new model, trained using both the initial manually labeled training set and unlabeled images with a prediction confidence of above 0.999, was then used to perform predictions on the remaining unlabeled images, resulting in a new round of pseudo-labeling. On this second round, the best threshold value found was 0.999999, achieving a validation accuracy of 94.66%, as shown in Fig. 5. After performing the same procedure two more times, the model resulting from the third round of pseudo-labeling used a total of 21,667 images for training (2354 manually labeled and 19,313 pseudo-labeled), and the model resulting from the fourth round of pseudo-labeling used a total of 22,418 (2354 manually labeled and 20,064 pseudo-labeled). The final model achieved an accuracy of 95.25% on the validation set and 92.71% on the test set, consisting of a 15.17% absolute and 19.6% relative increase on testing accuracy when compared to the original model trained using just the manually labeled images. These results show the great potential for improving the predictive performance of trained neural networks by using this relatively simple pseudo-labeling technique to leverage the information contained in large unlabeled image datasets.

**Figure 6:**
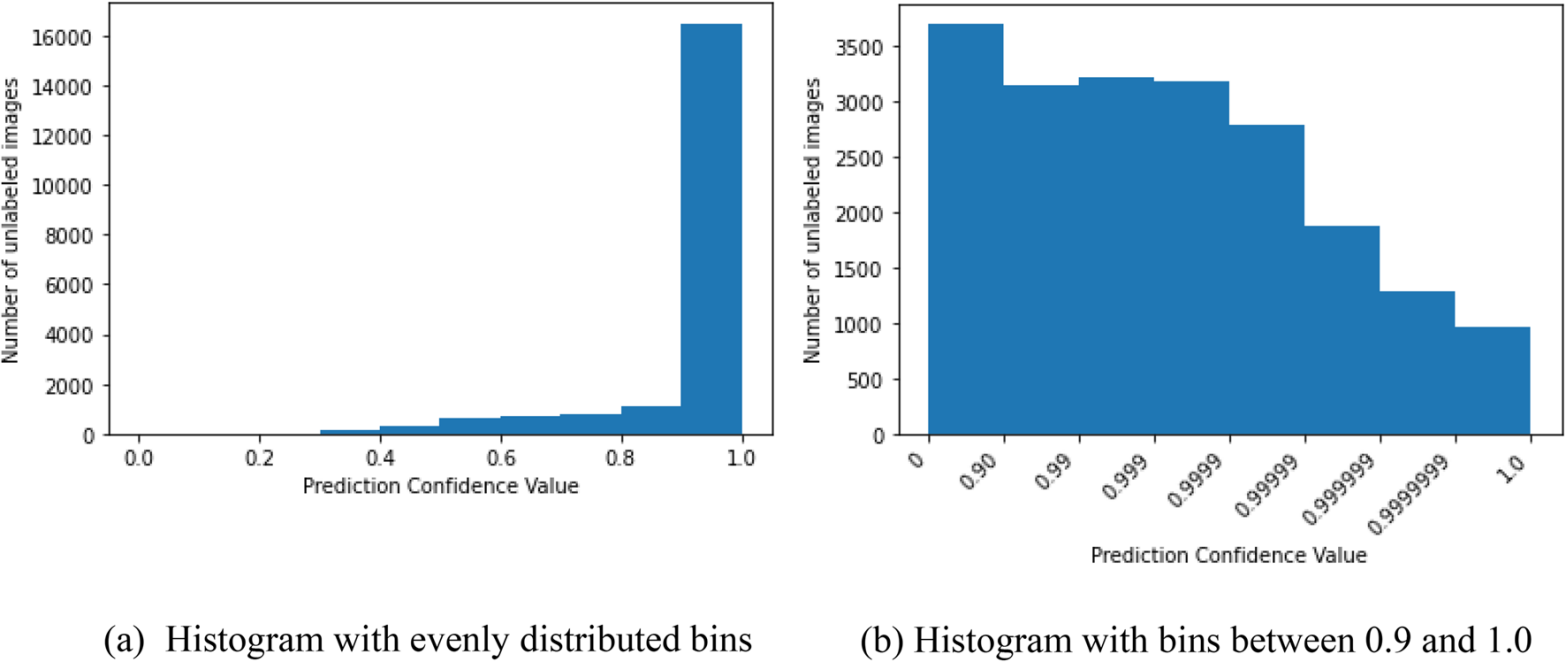
Distribution of the confidence values predicted by the baseline fully supervised Xception model on the unlabeled dataset illustrated through a histogram containing evenly distributed bins of size 0.1 (**a**), and through a histogram with bins between 0.9 and 1.0 to better indicate the distribution of confidence values closer to 1 (**b**). Although not numerically equal, bins in (**b**) were set to visually have equal widths for illustration purposes.

In the current study, similarly to^29^, we used a thresholding parameter to select which unlabeled images would be used for training, however, their corresponding pseudo-labels were not updated dynamically during training. Instead, the pseudo-labels were only updated after each full round of training, including new unlabeled images as their corresponding prediction confidences reached a value above the defined threshold. Defining the range of thresholds to be tested during hyperparameter tuning required a careful evaluation of prediction confidences in the unlabeled dataset. Threshold values that differed only after the 5th decimal place, for example, still resulted in significant performance and training set size differences, as seen in Figs. 3 and 4. Figure 6 illustrates histograms of the confidence values predicted by the baseline fully supervised Xception model on the unlabeled dataset.

As described in *Initial labeled training set size*, the same 4-round procedure was repeated starting with reduced baseline datasets. The original manually labeled training set was reduced to 10%, 25%, 50%, 75%, and 90% of its size, maintaining class proportions. Then, the accuracy on the same fixed testing set was evaluated at the end of the fourth round and compared to the test accuracy before performing pseudo-labeling. The results are shown in Table 3. Even when reducing the starting training dataset to 50% of its original size, the network resulting after the end of four rounds of pseudo-labeling can achieve a predictive performance better than that of the full manually labeled dataset. Even on very small labeled datasets (average of 20 images per cow, which corresponds to approximately 5% of the total number of labeled and unlabeled images dedicated to training), performing this pseudo-labeling technique can still significantly improve the accuracy of the trained neural networks.

**Table 3:** Training set size, test set accuracy, and percentage of images utilized (considering manually labeled and unlabeled images dedicated for training) before any pseudo-labeling and after performing four rounds of pseudo-labeling.

Dataset	Baseline training set size	Baseline test accuracy (%)	Final training set size	Final test accuracy^a^ (%)	% Test accuracy increase (%)	Initial % of images utilized (%)	Final % of images utilized (%)
10%	233	33.6	14,424	42.2	26	1	64
25%	585	51.5	18,972	71.9	40	3	84
50%	1177	70.9	21,138	89.7	27	5	94
75%	1769	71.4	21,920	89.7	26	8	97
90%	2123	74.2	22,280	91.3	23	9	99
full	2354	77.5	22,418	92.7	20	10	99

Even starting with as few as 5% of the total images available, performing pseudo-labeling allowed for up to 94% of the images to be retrieved, labeled, and used for training. The resulting neural networks achieved a relative increase in accuracy between 20 and 40% when compared to the networks trained without pseudo-labeling.

^a^Test accuracy after performing four rounds of pseudo-labeling.

## Conclusion

The main goal of this study was to present a new perspective to approach the problem of animal identification using computer vision—using semi-supervised learning. We evaluated the potential of a relatively simple semi-supervised learning technique called pseudo-labeling to improve the predictive performance of neural networks trained to identify individual Holstein cows. The method evaluated in this study is complementary to current animal identification research, as it can be seamlessly applied to previously trained models without requiring modifications in the model architecture or optimization procedure. We believe this use-inspired research highlights the potential of the evaluated method as a tool for advancing the field of animal identification, as it could be applicable for both closed- and open-set problems. Subsequent research could focus on comparing the evaluated method with other semi-supervised learning techniques, including those involving retraining a model from scratch. Furthermore, there is room for proposing modifications to existing SSL methods, tailoring the algorithms specifically to the task of animal identification. Additionally, it would be interesting to explore the efficacy of SSL techniques in the open-set scenario, as it reflects a more realistic setting for dynamic commercial herds.

## Data Availability

The datasets generated during and/or analyzed during the current study are available in the *Pseudo-labeling for animal identification* Open Science Foundation repository, https://osf.io/vyh5j/.
